# marmap: A Package for Importing, Plotting and Analyzing Bathymetric and Topographic Data in R

**DOI:** 10.1371/journal.pone.0073051

**Published:** 2013-09-03

**Authors:** Eric Pante, Benoit Simon-Bouhet

**Affiliations:** Littoral, Environnement et Sociétés Joint Research Unit 7266 Centre National de la Recherche Scientifique, Université de La Rochelle, La Rochelle, France; NASA Jet Propulsion Laboratory, United States of America

## Abstract

In this communication we introduce marmap, a package designed for downloading, plotting and manipulating bathymetric and topographic data in R. marmap can query the ETOPO1 bathymetry and topography database hosted by the NOAA, use simple latitude-longitude-depth data in ascii format, and take advantage of the advanced plotting tools available in R to build publication-quality bathymetric maps. Functions to query data (bathymetry, sampling information…) are available interactively by clicking on marmap maps. Bathymetric and topographic data can also be used to calculate projected surface areas within specified depth/altitude intervals, and constrain the calculation of realistic shortest path distances. Such information can be used in molecular ecology, for example, to evaluate genetic isolation by distance in a spatially-explicit framework.

## Introduction

Marine ecologists still lack simple, highly-customizable tools for building and using marine charts, open-source tools being particularly lacking. Currently, apart from commercial solutions, marine ecologists can use GIS tools such as GRASS GIS [1], or the graphical user interface provided by GeoMapApp [2] and Google Earth [3] to prepare publication-quality bathymetric charts, but these tools either require knowledge in the field of GIS, or are limited in the number of functions, datasets, and analyses available. The R environment [4] is a convenient platform for generating maps, thanks to its built-in functions for data matrix manipulation and advanced graphics. The package marmap [5], introduced herein, takes advantage of these built-in functions, as well as packages for spatial analysis developed by others, to provide a simple and flexible tool for importing, manipulating, plotting and exporting bathymetric data. It provides a simple platform for bathymetric data analysis and plotting even with limited experience with R, and allows production of publication-quality maps. The entirety of the marmap package was coded in the R language, providing advanced R users with the opportunity to fine-tune or expand any function. Also, because all bathymetric data are imported as R objects, the arsenal of R tools can be utilized for analyses not implemented in the marmap package.

## Description of the marmap Package

### Portability

Binaries and source code for the marmap package are freely available on the Comprehensive R Archive Network (CRAN [6]), along with a tutorial (also called “R vignette”). marmap contains R code exclusively, which maximizes its portability across platforms. CRAN makes the source code and binaries available for Unix-like and Windows operating systems [5].

### Importing and Plotting Bathymetric Data

marmap offers different ways to upload bathymetric information, which should provide flexible data preparation, depending on need and access to online resources. First, bathymetric data can be downloaded directly from within R by querying the ETOPO1 database [7] hosted by the NOAA. Second, three-column data frames (with longitude, latitude, and depth data) can be imported from different sources, such as the US National Ocean and Atmospheric Administration (NOAA) [8]. Third, marmap has functions to prepare and use a local SQL database, stored on the user's hard drive. Use of a personalized SQL bathymetry database is convenient for querying subsets of very large bathymetric datasets such as the 5Go ETOPO1. Finally, data can be imported like any other dataset into R, and converted using marmap functions. While marmap was originally designed for marine ecologists, it is fully compatible with topographical data, which can be treated along with bathymetric data, or analysed on their own.

**Figure 1:**
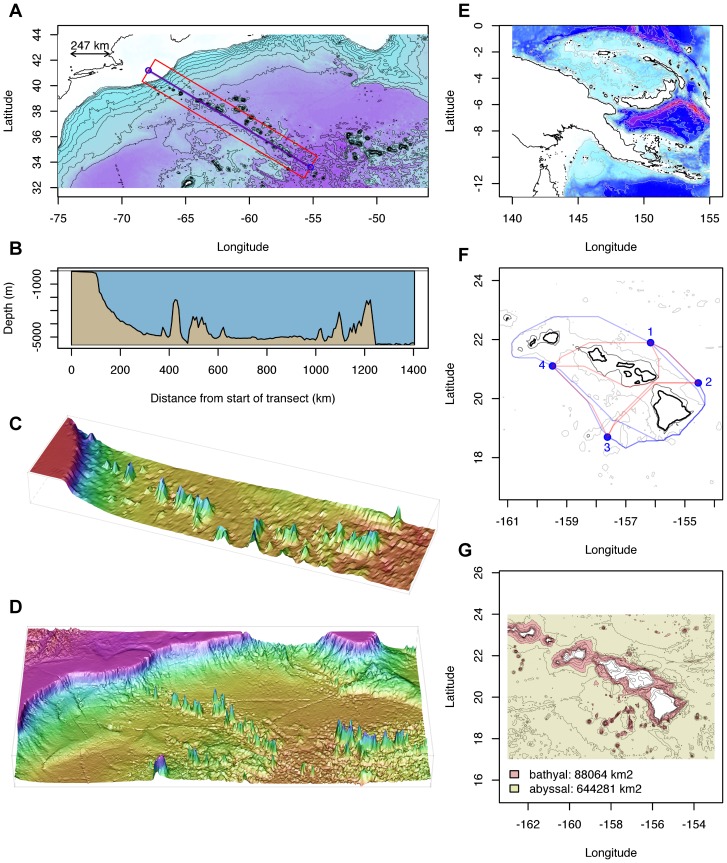
Examples of two and three dimensional plots created using marmap tools. Left panel (**A**): data from the NW Atlantic Ocean, showing the NE coast of the USA and the New England and Corner Rise seamounts chains. The blue line represents the position of two- (**B**) and three- (**C**) dimensional cross sections, the red rectangle delimiting the area covered by the belt transect. The bottom left figure (**D**) represents the NW Atlantic data as plotted with the wireframe function from package lattice [21], based on data imported with marmap. Right panel: map of Papua New Guinea and satellite islands (**E**; see text). The middle figure (**F**) represents the results of a least-cost path analysis around the Hawaiian islands (coastline in thick black, dark grey: 1000 m isobath, light grey 4000 m isobath; red line: path avoiding waters shallower than 1000 m, blue line: path avoiding waters shallower than 4000 m). The bottom figure (**G**) represents the results of projected surface area calculations for the bathyal and abyssal areas around the Hawaiian islands. R code is available in File S1.

Once loaded into R, bathymetric data are re-organized as a matrix that is used for manipulation, plotting and exporting. These data are represented as an R class called “bathy”, valid within a marmap session. The creation of a custom R class allows the use of generic functions such as plot and summary. Bathymetric data of class “bathy” can be plotted with the streamlined marmap plotting tools, or used with other geographic analysis packages (e.g. “maps” [9]). Bathymetric data can be plotted as simple contour plots, with control over the range, density and look of isobaths, or with automatic isobath choice and placement. Contour plots can be coupled with heat maps with built-in and customizable color ramps (Figure 1A,E). Sampling data (GPS points, tracks, polygons, etc…) and text (legends, labels, scales, etc…) can simply be added to marmap maps using R low-level functions.

For example, creating a map of Papua New Guinea can be done in three lines of code, in a new R session:

> library(marmap)
> getNOAA.bathy(lon1 = 140,lon2 = 155,lat1 = −13,lat2 = 0, resolution = 1) ->papoue
>plot(papoue, image = TRUE)

The first line loads marmap, the second queries bathymetric data (resolution of 1 minute) and stores the result in a variable of class “bathy,” and the third line creates a map with automatic isobath choice and built-in color ramp. The upper right map on Figure 1, plotted with isobaths of different widths and colors as well as a custom color ramp was produced with these two lines (the first creating and storing a custom palette, and the second creating the map; details on commands and their arguments are available in the marmap documentation on CRAN):

> colorRampPalette(c("red","purple","blue","cadetblue1","white")) -> blues
>plot(papoue, image = T, bpal = blues(100),
deep = c(−9000, −3000,0), shallow = c(−3000, −10, 0), step = c(1000, 1000, 0),
col = c("lightgrey","darkgrey","black"), lwd = c(0.3,0.3,0.6), lty = c(1,1,1),
drawlabel = c(F,F,F))

### Using Bathymetric for Further Ecological Analysis

By interactively clicking on a map, the user can retrieve bathymetric data (either from a point or an area), sampling information (e.g. list of specimens sampled within a particular geographic area), or two- and three-dimensional depth cross-sections (Figure 1B–C). Projected surface areas can also be estimated for specified depth ranges, such as the bathyal and abyssal zones around Hawaii (Figure 1G).

Based on functions developed by Jacob van Etten in the raster and gdistance packages [10], [11], marmap facilitates analysis of geographically-explicit ecological data, by allowing least-cost path calculation constrained by bathymetry and/or topography. Shortest great-circle (haversine) distance between pairs of sites, avoiding land masses or depth layers, are calculated. Outputs of least-cost path analysis can be in the form of great-circle distance, taking into account the curvature of the Earth, and in the form of geographical paths that can be plotted on marmap maps, and used to compute two- and three-dimensional depth cross-sections. Distance data can be easily exported for direct use in landscape genetics software such as TESS [12]. Data can also be used directly from within R to perform Mantel tests or other analyses [13]–[15].

### Interactions with other Packages

**Figure 2:**
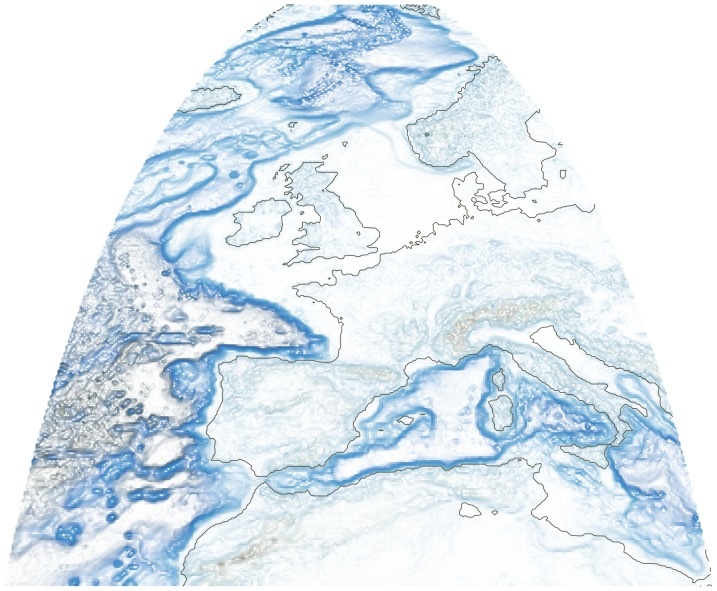
Exemple of ETOPO1 data downloaded with the marmap package, transformed with the raster package, and plotted with marmap. Orthographic projection, resolution of 10 minutes. R code is available in File S1.

marmap interacts with multiple existing R packages for visualization and analysis, such as lattice for building three-dimensional plots (Figure 1C–D), and gdistance for least-cost path calculations (see above). marmap also contains functions to ease interactions with other packages dedicated to the analysis of spatial data. Data from class bathy can be transformed into raster objets for use in the raster package [10] (functions trans.mat and as.raster). Bathy objects can also be converted into the class SpatialGridDataFrame for use in the sp package [16], [17] (function as.SpatialGridDataFrame). These transformed objects can be converted back into objects of class bathy using the as.bathy function. In the example below, the package raster is used to change the projection of a dataset extracted from ETOPO1. The first line creates a raster object from a bathy object, the second defines the target projection (PROJ.4 format), the third creates a new projected raster object, and the last converts the new raster projection into a bathy object for plotting with marmap. Figure 2 provides an example of such transformed data.

> r1<- as.raster(original.marmap.data)
> new.projection <- "+proj = ortho"
> r2<- projectRaster(r1,crs = new.projection)
>projected.marmap.data <- as.bathy(r2)

## Conclusions

marmap was designed to provide easy-to-use tools for importing and using bathymetric and topographic data in R. While marmap has been primarily designed for research, and the rapid production of publication-quality maps (examples: [18]–[20]), its simplicity of use should make it interesting as a teaching tool as well.

## Supporting Information

File S1 **R code used to produce** **Figures 1** **and** **2** **.**
(R) Click here for additional data file.
